# Altered levels of variant cholinesterase transcripts contribute to the imbalanced cholinergic signaling in Alzheimer’s and Parkinson’s disease

**DOI:** 10.3389/fnmol.2022.941467

**Published:** 2022-09-02

**Authors:** Muslum Gok, Nimrod Madrer, Tamara Zorbaz, Estelle R. Bennett, David Greenberg, David A. Bennett, Hermona Soreq

**Affiliations:** ^1^Department of Biochemistry, Faculty of Medicine, Muğla Sıtkı Koçman University, Muğla, Turkey; ^2^Edmond and Lily Safra Center for Brain Sciences, The Hebrew University of Jerusalem, Jerusalem, Israel; ^3^Department of Biological Chemistry, The Hebrew University of Jerusalem, Jerusalem, Israel; ^4^Biochemistry and Organic Analytical Chemistry Unit, Institute for Medical Research and Occupational Health, Zagreb, Croatia; ^5^Department of Neurological Sciences, Rush University Medical Center, Chicago, IL, United States

**Keywords:** Alzheimer’s disease, Parkinson’s disease, acetylcholinesterase, butyrylcholinesterase, SNPs (single-nucleotide polymorphisms), splice variants

## Abstract

Acetylcholinesterase and butyrylcholinesterase (AChE and BChE) are involved in modulating cholinergic signaling, but their roles in Alzheimer’s and Parkinson’s diseases (AD and PD) remain unclear. We identified a higher frequency of the functionally impaired BCHE-K variant (rs1803274) in AD and PD compared to controls and lower than in the GTEx dataset of healthy individuals (*n* = 651); in comparison, the prevalence of the 5′-UTR (rs1126680) and intron 2 (rs55781031) single-nucleotide polymorphisms (SNPs) of BCHE and ACHE’s 3′-UTR (rs17228616) which disrupt AChE mRNA targeting by miR-608 remained unchanged. qPCR validations confirmed lower levels of the dominant splice variant encoding the “synaptic” membrane-bound ACHE-S in human post-mortem superior temporal gyrus samples from AD and in substantia nigra (but not amygdala) samples from PD patients (*n* = 79, *n* = 67) compared to controls, potentially reflecting region-specific loss of cholinergic neurons. In contradistinction, the non-dominant “readthrough” AChE-R mRNA variant encoding for soluble AChE was elevated (*p* < 0.05) in the AD superior temporal gyrus and the PD amygdala, but not in the neuron-deprived substantia nigra. Elevated levels of BChE (*p* < 0.001) were seen in AD superior temporal gyrus. Finally, all three ACHE splice variants, AChE-S, AChE-R, and N-extended AChE, were elevated in cholinergic-differentiated human neuroblastoma cells, with exposure to the oxidative stress agent paraquat strongly downregulating AChE-S and BChE, inverse to their upregulation under exposure to the antioxidant simvastatin. The multi-leveled changes in cholinesterase balance highlight the role of post-transcriptional regulation in neurodegeneration. (235)

## Introduction

Alzheimer’s disease (AD) and Parkinson’s disease (PD) are chronic, age-related neurological disorders that involve impaired cognitive abilities and cognitive deficits in executive functions (Paul, 2021). AD primarily affects deep cholinergic nuclei (Schmitz et al., 2016), the hippocampus and the cortex, and leads to memory deterioration, whereas PD primarily affects motor functions, is characterized by loss of dopaminergic neurons in the substantia nigra, and involves large-scale brain transcriptional changes (Chen et al., 2019; Dinda et al., 2019; Hanan et al., 2020). Recent epidemiological studies indicate that worldwide, over 50 million individuals are affected by AD (Karlawish et al., 2017; Association, 2019) and 10 million by PD (Tysnes and Storstein, 2017; Vasili et al., 2019), with both these numbers increasing rapidly.

Acetylcholine (ACh) coordinates both peripheral and central nervous systems, and perturbations of ACh levels modify the dynamic balance between its synthesis and hydrolysis, altering what is termed “the cholinergic tone,” which may cause both brain and body pathology (Madrer and Soreq, 2020). The cholinergic tone is impaired in numerous neurodegenerative diseases, but how genes expressed in the brain respond to this imbalance is unclear (Silman, 2021). AD therapeutics, in use for several decades, primarily involve the administration of AChE inhibitors to prolong ACh availability at the synapse and thus improve AD-related symptoms (Campanari et al., 2016; Giacobini et al., 2022). Therefore, finding the cause of changes in the cholinergic tone is important. The effects of ACh are terminated by rapid hydrolysis, primarily by AChE and BChE (Soreq and Seidman, 2001; Mesulam et al., 2002). Hence, changes in AChE and/or BChE expression may alter the cholinergic tone.

The major AChE isoform is the membrane-bound synaptic AChE-S (AChE-T) isoform, with a less abundant C-terminal shorter soluble variant translated from the stress-induced “readthrough” variant AChE-R (Soreq and Seidman, 2001). In blood, the membrane-associated hydrophobic variant AChE-E (AChE-H) is bound to erythrocyte membranes via a GPI anchor (Grisaru et al., 1999). These three major alternative splice variants of AChE mRNA may be translated into AChE proteins, with an alternative upstream promoter yielding a rare AChE splice variant with an extended N-terminus (AChE-Next) (Meshorer et al., 2004). In comparison, BChE has only one mRNA and protein isoform (Soreq et al., 2021; Zorbaz et al., 2022). Furthermore, the AChE and BChE mRNA and protein levels are differentially regulated in distinct cell types and pathological situations and diseases. For example, AChE-S plays an important role in AD (Jing et al., 2013) and in inflammatory states (Vaknine and Soreq, 2020), while AChE-R mRNA and AChE-Next levels are higher in stress-related or neurodegenerative disorders (Meshorer and Soreq, 2006).

Amino acid-modifying mutations and single-nucleotide polymorphisms (SNP) in either the coding or non-coding regions of BChE or AChE transcripts can alter their mRNA levels or enzyme activities, changing the cholinergic tone (Lockridge, 2015; Jasiecki et al., 2019). This disrupts the ACh communication network in both brain and body, which may impair cognitive and body functioning. There are over 70 mutations in the BCHE gene, the most common of which is the BCHE-K variant with reduced hydrolytic activity (rs1803274) (Lockridge, 2015). In addition, SNPs in the non-coding 5′-untranslated region (5′-UTR) (rs1126680) and intron 2 (rs55781031) of the BCHE gene may play as yet undiscovered roles in the emergence and progression of AD and PD (Rösler et al., 2018; Jasiecki et al., 2019). Furthermore, microRNA (miRNA) regulators of cholinergic signaling (“CholinomiRs”) (Nadorp and Soreq, 2014) may play key roles in defining the cholinergic tone. Due to their small size, miRNAs can be synthesized quickly and alter entire biological pathways, enabling efficient and energy-saving regulation of the production of various proteins (Soreq, 2015). CholinomiRs are thus particularly well suited to regulate the rapidly adaptive physiology of the parasympathetic system. They could modulate both the neuronal and immunological functions of ACh by altering the cholinergic tone (Shaked et al., 2009; Simchovitz et al., 2017). In this context, a SNP in the 3′-untranslated region (3′-UTR) of ACHE (rs17228616) attenuates AChE mRNA’s interaction with the primate-specific miRNA-608, and homozygous carriers of this SNP display elevated anxiety and hypertension (Hanin et al., 2014).

**FIGURE 1:**
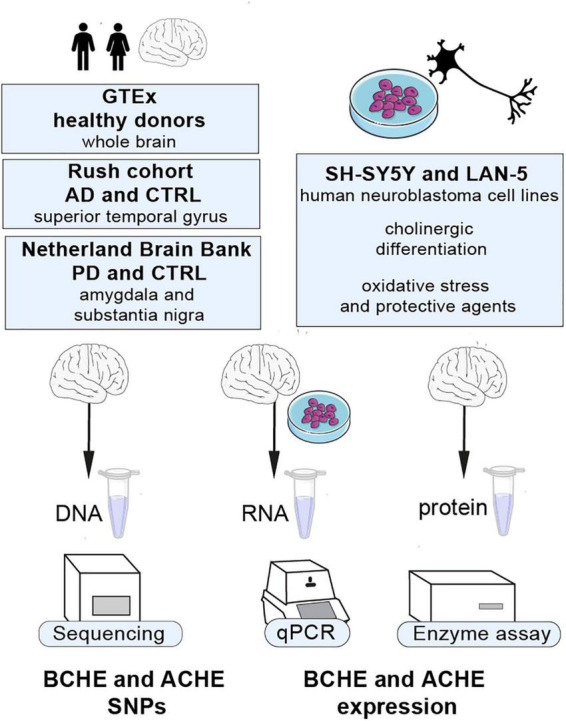
Our study’s outline. Cholinergic transcripts were analyzed by qPCR, Sanger sequencing, and enzyme activity assays from different regions of human post-mortem brains from AD and PD patients, SH-SY5Y and LAN-5 cells, and GTEx cohort datasets.

To study the link between changes in cholinergic signaling, both inherited and post-transcriptional, and the neurodegenerative diseases AD and PD, we used human post-mortem brains from AD (*n* = 79), PD (*n* = 67), and the GTEx dataset (*n* = 651) to search for co-inherited SNPs in both the coding and non-coding regions of the BCHE gene and in the 3′-UTR region of the ACHE gene. We also sought genetic variants and transcript levels of the BCHE gene and quantified alternatively spliced AChE mRNA transcripts in the amygdala and substantia nigra of post-mortem brains from PD patients and in the superior temporal gyrus of AD brains, both compared to apparently healthy control brains. Both the substantia nigra as a basal ganglia movement control center, and the amygdala as a center of emotions and memories, are regions with specific pathological findings in PD, while the superior temporal gyrus is part of the auditory cortex involved in speech recognition and damaged in AD. Therefore, we anticipated the existence of disease- and brain region-specific changes in splice variants and activity of cholinesterases in both these cases. We used human-derived neuroblastoma cell lines (SH-SY5Y and LAN-5) to seek changes in the cholinesterase splice variants under cholinergic differentiation and following exposure to an oxidative stress-inducing agent or to an antioxidant. Our findings identify specific changes in the levels and composition of cholinesterase transcripts in the context of both AD and PD and in cultured neuronal cells of human origin under different conditions (see Figure 1 for an outline of this study).

## Materials and methods

### Brain tissues

Two sets of frozen human post-mortem brain tissues were provided by the Rush Alzheimer’s Disease Center (*n* = 79) and by the Netherland Brain Bank (NBB; *n* = 67), accompanied by clinical and pathological evaluations (Barbash et al., 2017; Bennett et al., 2018; Simchovitz et al., 2019; Hanan et al., 2020). Donors of our tested AD tissues showed either no cognitive impairment (NCI), mild cognitive impairment (MCI), or AD dementia (DEM), each cognitive category containing three cases with low (Braak 1–2), moderate (Braak 3–4), and high (Braak 5–6) plaque scores (Barbash et al., 2017). The use of tissues was approved by the Netherland Brain Bank, the Rush University Medical Centre Institutional Review Board, and the Hebrew University’s Ethics Committee (for a complete list of samples, see Supplementary Tables 1, 2). All of these samples were derived from donors for whom or from whom NBB and the Rush Religious Orders Study had obtained written informed consent to autopsy the brain and to use the material and clinical information for research purposes.

### RNA extraction from human brain and cell culture samples

The amygdala and substantia nigra of PD post-mortem brains and the superior temporal gyrus from brains of AD patients, both accompanied by matched controls, were used for total RNA extraction. To preserve RNA integrity, tissue pieces of 30–50 mg were cut on dry ice to ensure they did not defrost, then homogenized in 700 μl of QIAzol Lysis Reagent, and RNA was extracted according to the manufacturer’s protocol (miRNeasy Kit, Qiagen, Germantown, MD, United States). NanoDrop (Thermo Fisher Scientific, Waltham, MA, United States) was used to determine RNA concentration, and gel electrophoresis was performed to assess RNA quality.

### Single-nucleotide polymorphisms genotyping of human brain tissues

DNA was extracted from AD and PD human post-mortem brain tissues according to the manufacturer’s protocol (The DNeasy Tissue Kit, Qiagen, Germantown, MD, United States). PCR was used to amplify three regions of the BCHE gene, using the primers listed in Supplementary Table 3. The automated Sanger DNA sequencing method used the BigDye Terminator Cycle Sequencing Kit from Applied Biosystems (Thermo Fisher Scientific, Waltham, MA, United States), with final concentrations of 0.4 ng/μl DNA and 0.3 μM primer. Samples were sequenced using the 96-capillary DNA analyzer 3730xl and the ABI software served for data acquisition and sequence analysis. Sequences were analyzed and aligned with the BCHE genomic reference sequence using the Integrative Genomics Viewer (IGV) 2.11 software. In addition, patients’ genotypes were derived from the Genotype-Tissue Expression Database for GTEx patients or verified by Sanger sequencing for available AD and PD brain tissues. FDR-corrected association and linkage between all three variant combinations were determined using the chi-square test.

### Cell culture experiments

Human-derived SH-SY5Y (ATCC CRL-2266) and LAN-5 (DSMZ ACC 673) neuroblastoma cells were grown per ATCC instructions and as described (Lobentanzer et al., 2020) at 37°C and 5% CO_2_ in EMEM: F-12 (SH-SY5Y) and RPMI 1640 (LAN-5), both containing FCS (10% final concentration, 04-127, Sartorius), L-glutamine (2 mM final concentration, 03-020, Sartorius), and penicillin–streptomycin–amphotericin (100 units/ml, 0.10 mg/ml, 0.25 μg/ml, final concentrations, respectively, 03-033, Sartorius).

For cholinergic differentiation, we developed a re-optimized protocol. Briefly, LAN-5 and SH-SY5Y cells were seeded in 12-well plates at 100 000/well and, after 24 h, were treated with 100 ng/ml CNTF (recombinant human ciliary neurotropic factor, PeproTech, 450-13) and 10 μM ATRA (all-trans retinoic acid, Merck, R2625), while cells treated with 0.1% DMSO served as controls. Four days later, the medium was removed and cells were washed with cold PBS and then collected with 700 μl QIAzol (QIAGEN, 217004). Differentiation was assessed by measuring the levels of transcripts encoding for choline acetyltransferase (ChAT) and vesicular acetylcholine transporter (VAChT; SLC18A3) or high-affinity choline transporter (ChT; SLC5A7) as mRNA markers of cholinergic differentiation.

In the paraquat exposure (PQ) experiments, SH-SY5Y cells were incubated with 25 μM and 50 μM PQ (Sigma) concentrations in complete media for 24 h at 37°C or treated with 10 μM simvastatin compared to non-treated cells. For RNA analysis, medium was aspirated, wells washed with PBS, and cells lysed and homogenized with 700 μl of QIAzol. RNA was extracted using the miRNeasy kit and then treated with Ambion DNAse according to the manufacturer’s instructions (Thermo Fisher Scientific, Waltham, MA, United States). For mRNA quantitation, cDNA was prepared using the Quanta qScript mRNA cDNA Synthesis Kit (Quantabio, Beverly, MA, United States) according to the manufacturer’s instructions and diluted 1:10 in double-distilled water before setting up of qPCR plates (either 384-well or 96-wells) and running on a CFX-384/96 instrument (Bio-Rad) using Perfecta Sybr Green FastMix with Low or No Rox (Quantabio) at final volumes of 5 or 15 μl, respectively. Glyceraldehyde-3-phosphate dehydrogenase (GAPDH) served as a housekeeping control gene for both human brain and SH-SY5Y tests with the addition of ubiquitin C (UBC) or beta-actin (ACTB) for samples of cholinergic differentiation experiments. Transcript levels were calculated as ΔΔCq values using Bio-Rad CFX Maestro 1.1 software (primer sequences are listed in Supplementary Table 3). The annealing temperature was 60°C for all primers. Serial dilution of samples served to evaluate primers’ efficiency and cDNA concentrations, resulting in linear Cq-concentration dependence. The absence of genomic DNA was verified with reverse transcriptase-free controls.

### Measuring acetylcholine hydrolysis activities

Before homogenization, samples were placed on ice, weighed rapidly, mixed with five-fold volumes of low salt detergent (LSD) lysis buffer (10 mM sodium phosphate buffer pH = 7.4, 1% Triton X-100), and homogenized with a pellet-pestle. To measure the ACh hydrolysis activity of cholinesterases, we adapted the Ellman spectrophotometric method (Ellman et al., 1961) for use in the 96-microplate assay. Hydrolysis rates of acetylthiocholine (ATCh, Sigma, 1 mM) were measured by adding 10 μl brain tissue homogenate to each well of the microtiter plates for 20 min of preincubation with 5 × 10^–5^ M iso-OMPA (Sigma), a specific BChE inhibitor, in sodium phosphate buffer (0.1 M pH 7.4, 0.15 mM DTNB). ATCh hydrolysis rate was measured in the presence or absence of iso-OMPA to yield AChE activity and total activity of both AChE and BChE, respectively. Subtraction of AChE from total activity provided calculated BChE activity. Readings at 405 nm were repeated at 1-min intervals for 20 min using a Spark 10 M microplate reader (Tecan, Männedorf, Switzerland). Non-enzymatic degradation of the substrate was subtracted from the total hydrolysis rate. Enzyme activities were calculated using an extinction coefficient of 13,600 M^–1^ cm^–1^ for 2-nitro-5-thiobenzoate at 405 nm. Protein concentration of samples was determined by the Lowry method (Lowry et al., 1951) using a 96-well plate DC™ Protein Assay Kit (Bio-Rad, CA, United States). Absorbance values were measured at 750 nm using a Spark 10 M microplate reader. All assays were performed in triplicates.

### Statistical analyses

Statistics for donor samples are shown in boxplots, and statistics for cell culture samples are shown in bar graphs. All comparisons of expression were assessed by unpaired Welch’s test, and FDR multiple comparisons were accounted for. Frequency comparisons were conducted using chi-square test and were FDR-corrected. Linkage disequilibrium was assessed using the LDpair tool of the NIH LD link (Machiela and Chanock, 2015).

## Results

### Higher prevalence of the BCHE-K allele in Parkinson’s diseases compared to controls and linkage of the three BCHE single-nucleotide polymorphisms

**FIGURE 2:**
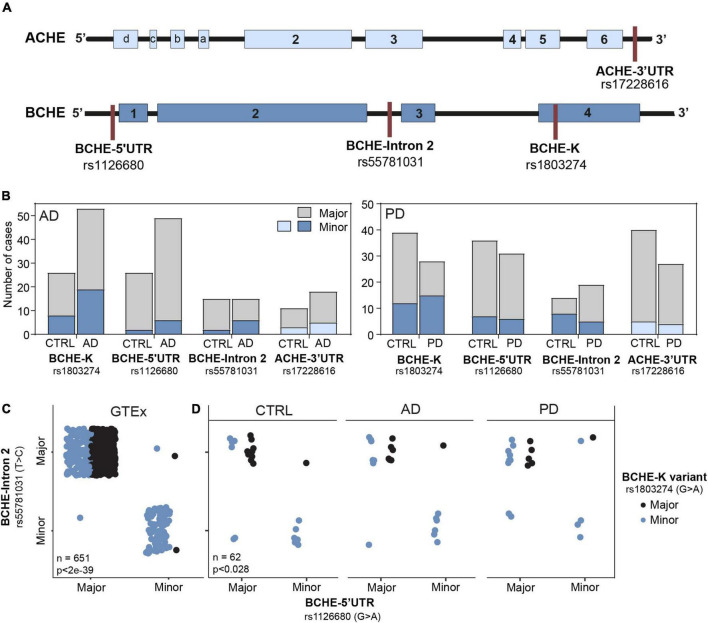
Altered prevalence of ACHE and BCHE SNPs in AD and PD patients compared to non-demented controls. **(A)** Loci of SNPs in the BCHE (K allele, rs1803274), 5′-UTR (rs1126680), and intron 2 (rs55781031), and of 3′-UTR ACHE SNP (rs17228616). SNPs were studied in AD (*n* = 79) and PD (*n* = 67) patients and in the genomic dataset of the GTEx healthy donors (*n* = 651). **(B)** The SNPs in the 5′-UTR (rs1126680) and intron 2 (rs55781031) of the BCHE gene, as well as the 3′-UTR ACHE SNP (rs17228616), showed similar occurrence in the diseased and healthy cohorts of AD and PD. **(C)** In the GTEx cohort, carriers of the 5′-UTR BCHE SNP (rs1126680) tended to carry the BCHE SNP in intron 2 (rs55781031) but did not show distinct prevalence of the coding sequence BCHE-K allele (rs1803274). **(D)** Both AD and PD donors of our small cohorts showed higher prevalence of the BCHE-K minor allele (rs1803274) compared to controls.

First, we searched for differences in allelic frequencies among both the coding and non-coding sequences of the ACHE and BCHE genes (Figure 2A) in non-demented controls (CTRL), AD, and PD as compared to the apparently healthy donors of the GTEx cohort. To this end, we sought the prevalence of SNPs in the 3′-UTR of the ACHE gene or single and dual co-inherited SNPs in coding or non-coding regions of BChE in AD (*n* = 69), PD (*n* = 28), and CTRL (*n* = 54) brains as compared to the genetic dataset of the GTEx cohort (*n* = 651). We found the BCHE-K variant (rs1803274) to be less common in CTRL and AD patients in comparison with the GTEx cohort (Table 1). Inversely, the BCHE-Intron 2 variant (rs55781031) was found to be more common in CTRL and AD compared to the GTEx cohort (Table 1 and Figure 2B). We then compared the prevalence of these SNPs within the Rush and NBB cohorts. Sixteen patients carried all three BCHE mutations, compatible with previous studies which showed structural differences in the BCHE-K protein (Podoly et al., 2009). Briefly, patients B1, B31, B50, B56, A16, and A55 (Supplementary Table 2) in the AD cohort as well as 10/67 PD patients carried all three BCHE mutations, but there was no significant association between these changes and the disease stages in either of these small cohorts.

**TABLE 1:** Control, AD, and PD groups show distinct distribution of the three BCHE SNPs from GTEx healthy donors.

Group	SNP	Observed ratio	Expected ratio	*P-value*	FDR
CTRL	BCHE-K	0.4	1.9	2e-08	2e-07
PD	(rs1803274)	1.2		0.204	0.329
AD		0.6		2e-06	1e-05

CTRL	BCHE-5′UTR	0.2	0.2	0.604	0.703
PD	(rs1126680)	0.3		0.219	0.329
AD		0.1		0.644	0.703

CTRL	BCHE-Intron 2	0.6	0.2	4e-04	2e-03
PD	(rs55781031)	0.4		0.092	0.220
AD		0.5		0.004	0.012

CTRL	ACHE-3′UTR	0.2	0.2	0.588	0.703
PD	(rs17228616)	0.2		0.971	0.971
AD		0.4		0.112	0.224

The table shows the observed ratio between the major and minor alleles in the AD and PD datasets compared to the expected ratio was the distribution identical to that of the GTEx dataset. P-values are derived from the chi-square test.

Subjects who carried all three BCHE SNPs at high allele frequency in the AD cohort include demented patients B50 and B56 with high Braak score, B31 and B1 with low Braak score, and subjects A16 and A55 with mild cognitive impairment (MCI) and high and low Braak score, respectively. Carriers of all three BCHE SNPs in the PD cohort included donor numbers 6, 14, 16, 45, 46, 50, and 152 from the non-demented controls and 125, 139, and 163 from the demented PD patients (Supplementary Table 1).

Intriguingly, when comparing each of the SNPs separately, we found BCHE-K to be more abundant in PD patients than in AD patients and CTRLs (54% of the PD and only 28% of the CTRL patients had the minor allele, *p* < 0.04, chi-square test, FDR; Figure 2D). All other SNPs [5′-UTR (rs1126680) and in intron 2 (rs55781031) of the BCHE gene and in the 3′-UTR of the ACHE gene (rs17228616); Figure 2B] showed similar occurrence in the diseased and healthy cohorts of AD and PD (Figure 2D). We also tested for linkage disequilibrium (LD) between the ACHE and BCHE SNPs to AD-related APOE ε4. While none of the BCHE SNPs showed significant LD, the ACHE SNP presented significant LD with APOE ε4 (rs429358: *D* = 0.0938, *p* < 0.0001; rs7412: *D* = 0.0434, *p* < 0.0065) in a separate dataset of 5,008 patients (Machiela and Chanock, 2015). Furthermore, in all of the tested populations, carriers of the 5′-UTR BCHE SNP (rs1126680) were most likely to also carry the BCHE SNP in intron 2 (rs55781031), reflecting co-inheritance of these two SNPs (GTEx cohort: *p* < 8e-136, NBB and Rush cohort: *p* < 6e-7, chi-square test, FDR; Figures 2C,D). BCHE-K was also found to be linked to the other two SNPs in that gene; patients carrying the BCHE-K major allele were highly probable to carry the major alleles of the two other SNPs as well (GTEx: *p* < 2e-39 NBB and Rush cohort *p* < 0.028, chi-square, FDR; Figures 2C,D). LD analysis on a separate cohort (*n* = 5,008) (Machiela and Chanock, 2015) further strengthened the connection between the currently studied SNPs and showed that rs1803274 is in significant LD with rs1126680 (*D* = 0.8913, *p* < 0.0001) and with rs55781031 (*D* = 1, *p* < 0.0001). SNPs rs55781031 and rs1126680 are further found to display significant LD with each other (*D* = 0.98, *p* < 0.0001). On the contrary, of all BCHE SNPs, only rs1803274 (K variant) presented a trend for significant LD with the ACHE SNP (*D* = 0.03, *p* < 0.052). Interestingly, BCHE-K was also more abundant in the Irish population (McIlroy et al., 2000) compared to the GTEx cohort of apparently healthy donors (*n* = 651) (Figure 2C), possibly indicating population differences as a reason. We conclude that globally measured BChE activities may be lower in brains of AD patients due to the higher incidence of the less hydrolytically active BCHE-K allele which may merely reflect population differences.

### Different levels of cholinesterase transcripts in Alzheimer’s disease and Parkinson’s diseases brains

A balance between ACh production and degradation (termed “cholinergic tone”) contributes to the dynamics of ACh levels and depends, to a certain extent, on the levels of the hydrolyzing enzymes AChE and BChE which in turn reflect their corresponding mRNA levels. Furthermore, AChE mRNA occurs in several alternative splicing variants, the leading one being the “synaptic” AChE-S. To address the expression levels of cholinesterase genes and their splice variants, we performed quantitative qPCR measurements using RNA from specified AD and PD brain tissues. Appreciating the need to only use high-quality RNA in transcriptome and mRNA analysis of human post-mortem brain tissue (Highet et al., 2021), we first verified tissue RNA integrity numbers (RIN) with the Agilent 2100 Bioanalyzer system and only employed samples with > 6.5 RIN values.

**FIGURE 3:**
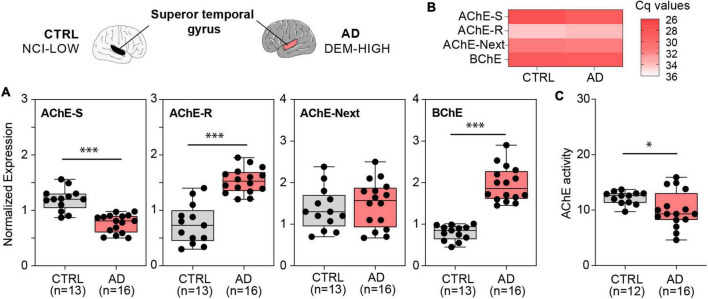
ACHE and BCHE mRNA transcripts and AChE enzyme activity show changed levels in the superior temporal gyrus of AD patients vs. controls. **(A)** Lower AChE-S mRNA (*p* < 4e-6), higher AChE-R mRNA (*p* < 3e-6). Unchanged AChE-Next mRNA, and higher BChE mRNA (*p* < 8e-9) in AD compared with controls, **(B)** qPCR-obtained Cq values for each cholinesterase show the predominance of the AChE-S splice variant over the AChE-R and AChE-Next transcripts in the superior temporal gyrus. **(C)** Decreased AChE enzyme activity (*p* < 0.05) in AD compared to control brains. Normalized expression represents ΔΔCq, and enzyme activity is shown in nmol ATCh/min/mg protein, ^∗^*p* < 0.05 and ^∗∗∗^*p* < 0.001.

From the AD cohort, we selected subjects with dementia and high Braak score (AD, DEM-HIGH) (*n* = 16) as the AD group and subjects with no cognitive impairment and low Braak score (CTRL, NCI-LOW) (*n* = 13) as controls. Quantitative qPCR results showed that the superior temporal gyrus from individuals with AD displayed different levels of both AChE and BChE mRNA transcripts compared to controls (Figure 3). Specifically, we observed lower AChE-S mRNA levels in AD brains compared to control ones (*p* < 0.001) (Figure 3A). By contrast, the mRNA levels of the “readthrough” AChE-R variant, known to be stress-induced, were higher in AD brains (*p* < 0.001) compared to controls (Figure 3A). Since AChE-R mRNA levels were very low compared to those of the major AChE-S transcript, both the total levels of catalytically active AChE and the levels of its transcripts were lower in the AD brains compared to controls (Figures 3A,C).

One of the least characterized AChE variants is the “N-terminus extended” AChE-Next variant, in which a 5′-encoded exon of AChE generates an alternative untranslated region and may give rise to a protein with an elongated N-terminus in humans, which is referred to as AChE-Next (Meshorer et al., 2004). We found similar AChE-Next mRNA levels in AD compared to control brains (Figure 3A). Finally, BChE acts as a substitute for AChE in ACh hydrolysis under crisis situations (Lockridge, 2015). Unlike AChE, there are no alternative splice variants of BChE in humans. The mRNA levels of BChE were higher in the AD brains, contrasting with the lower levels of AChE-S mRNA (Figure 3A). Also, measuring AChE’s enzyme activity revealed lower activity in AD brains compared with controls (*p* < 0.050), consistent with the observed decrease in AChE-S mRNA (Figure 3C), possibly indicating a contribution of brain-originated AChE to these brain measurements.

**FIGURE 4:**
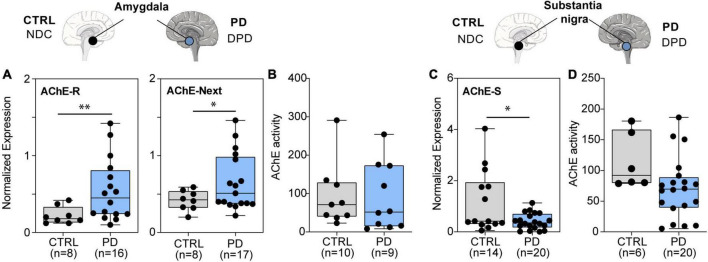
PD-modified levels of brain cholinesterase transcripts and AChE enzyme activity in the amygdala and substantia nigra. **(A)** Elevated mRNA levels of AChE-R (*p* < 0.023) and AChE-Next (*p* < 0.05) variants. **(B)** Unchanged AChE enzyme activity in PD compared with controls in the amygdala. **(C)** Decreased AChE-S mRNA levels (*p* < 0.05). **(D)** Decreased AChE enzyme activity (*p* = 0.078) in PD compared to controls in the substantia nigra. Normalized expression represents ΔΔCq, and enzyme activity is in nmol ATCh/min/mg protein, ^∗^*p* < 0.05, ^∗∗^*p* < 0.01.

Next, we quantified AChE-S mRNA levels in the amygdala (*n* = 25) and substantia nigra (*n* = 34) tissues from brains of demented PD patients (DPD = PD) and non-demented controls (NDC = CTRL). Both AChE-R and AChE-Next mRNA levels were elevated (*p* < 0.05) in the amygdala (Figure 4A), while AChE’s enzyme activity was unchanged (Figure 4B) and AChE-S mRNA transcripts presented a trend of decrease in the substantia nigra of PD compared to control donors (*p* < 0.1) (Figure 4C). However, there was no change in AChE-R or AChE-Next in the substantia nigra of PD brains compared to controls. Furthermore, hydrolytic AChE activity showed no disease-related change in the amygdala but was lower in the substantia nigra of PD brains compared to controls (*p* = 0.078) (Figure 4D). Likewise, BChE mRNA levels remained unchanged, and BChE enzyme activity was extremely low in the substantia nigra and amygdala from both PD and control brains. We identified altered levels of AChE and BChE mRNAs, and modified ACh hydrolytic activity in AD and PD brains reflects a primarily post-transcriptional cholinergic imbalance and highlights both disease- and brain area-specific changes in the post-transcriptional regulation of AChE variants.

### Cholinergic differentiation, oxidative stress, and antioxidant treatment shift the balance between cholinesterase splice variants in neuroblastoma cells

**FIGURE 5:**
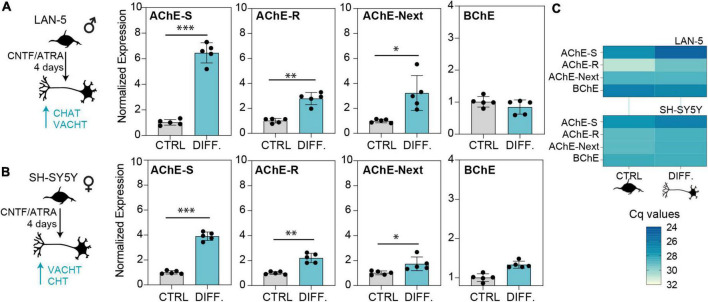
Cholinergic differentiation of neuroblastoma cell lines elevates the levels of AChE-S, AChE-R, and AChE-Next, but not BChE transcripts. Cholinergic differentiation induced by 4 days of treatment with CNTF and ATRA led to elevation of cholinergic markers CHAT, SLC18A3 (VAChT), and SLC5A7 (CHT) (Supplementary Figure 1). **(A)** Specifically, LAN-5 cells showed upregulation of all AChE variants (AChE-S, *p*-value < 0.0004; AChE-R, *p*-value < 0.004, AChE-Next, *p*-value < 0.02). **(B)** SH-SY5Y cells showed upregulated AChE-S (*p*-value < 0.0001), ACHE-R (*p* < 0.01), and ACHE-Next (*p* < 0.05). BChE mRNA levels remained unchanged in both cell lines. **(C)** RT-qPCR Cq values show the predicted predominance of AChE-S over BChE or AChE-R and AChE-Next transcripts in cholinergic-differentiated LAN-5 and SH-SY5Y cells. Normalized expression represents ΔΔCq. *T*-test *p*-values, ^∗^*p* < 0.05, ^∗∗^*p* < 0.01, and ^∗∗∗^*p* < 0.001.

Human brain cholinesterases are primarily expressed in neurons (Mesulam et al., 2002). The above shifts in the balance between the variants called for further exploring the regulation of individual cholinesterase variants in non-diseased neurons, for which purpose we employed cultured human-derived LAN-5 (Figure 5A) and SH-SY5Y (Figure 5B) neuroblastoma cells of male and female origin, respectively, as an *in vitro* model of cholinergic neurons’ differentiation. Both cell lines showed increases in all three variants of AChE, indicating their role in defining a cholinergic state. Expectedly, AChE-S mRNA showed the largest increase, while AChE-R and AChE-Next mRNA had less prominent elevations. BChE was unchanged in either cell line (Figures 5A–C), compatible with previous reports of brain BChE being mostly located in glial cells (Wright et al., 1993).

**FIGURE 6:**
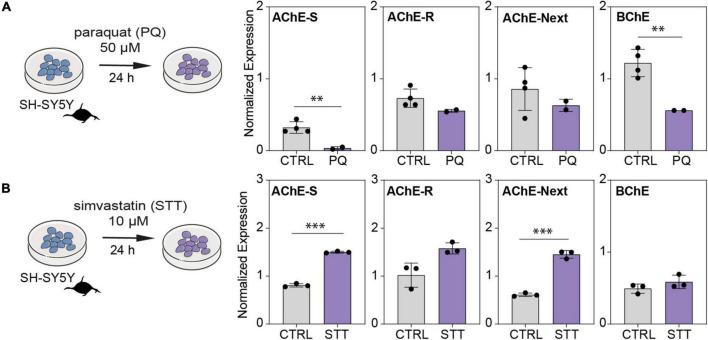
Oxidative stress and antioxidant treatment shift cholinesterase mRNA levels inversely in neuroblastoma cells. **(A)** SH-SY5Y cells were exposed to the oxidative stress-inducing agent paraquat (PQ; 50 μM) or to **(B)** antioxidant simvastatin (STT; 10 μM) for 24 h. Note PQ-induced decreases in AChE-S (*p* < 0.015), BChE (*p* < 0.015), AChE-R (*p* = 0.09), and AChE-Next (*p* = 0.234), and STT-induced increase in AChE-S (*p* < 0.0003), BChE (*p* = 0.23), AChE-R (*p* < 0.06), and AChE-Next (*p* < 0.002). Normalized expression represents ΔΔCq, ^∗^*p* < 0.05, ^∗∗^*p* < 0.01, and ^∗∗∗^*p* < 0.001.

To further challenge the impact of oxidative stress as a cellular mechanism inducing neuronal damage via dysregulation of the balance between cholinesterase variants, we exposed SH-SY5Y cells to the oxidative stress-inducing agent paraquat (PQ) and to the antioxidant simvastatin (STT). The AChE-S, AChE-R, AChE-Next, and BChE transcripts were all drastically reduced in cells exposed to PQ compared with control conditions (Figure 6A). In contrast, cells treated with the neuroprotective agent STT showed elevation of the AChE-S, AChE-R, AChE-Next, and BChE mRNA transcripts compared with control cells (Figure 6B). Thus, modulated cholinergic tone in cultured cholinergic cells of human neuronal origin may explain some of the changes observed in patients’ brain tissues.

## Discussion

Alzheimer’s disease and Parkinson’s disease are age-related neurodegenerative diseases with distinct pathological features characteristic of specific disease mechanisms (Nam et al., 2019; Vasili et al., 2019). Both diseases involve acute stress symptoms and neuroinflammation states, predicting relevant transcriptional changes. Seeking such changes in human post-mortem brain tissues is essential for understanding the pathogenesis of neurodegenerative diseases (Highet et al., 2021). We found disease-characteristic inherited and post-transcriptional differences of cholinesterase variants in post-mortem brain tissues from AD and PD patients which were consistent with the patients’ mental symptoms. This may indicate different transcriptional and brain cell death processes in AD and PD individuals with cognitive impairments.

Compatible with previous reports in AD and PD, we identified a higher occurrence of the BCHE-K variant together with SNPs in non-coding region of BCHE (rs1126680, rs55781031) as linked to high risk for AD (6 of 79 patients had all three BCHE SNPs). The BCHE-K variant also showed significantly higher occurrence in our small PD sample (15 of 28 patients, 54%) compared with controls (12 of 39 individuals, 30%). Others showed that carriers of all three BCHE SNPs and the ApoE4 allele were at higher risk for late-onset AD (10 out of 55 patients) (Jasiecki et al., 2019). In comparison, our AD samples (*n* = 79) showed no relevance of patients’ APOE alleles to late-onset AD prediction. Rather, both our AD and PD samples and the larger GTEx cohort displayed modified incidence of SNPs in both the coding and non-coding sequences of the BCHE gene and co-inheritance of the BCHE SNPs in the 5′-UTR (rs1126680) and intron 2 (rs55781031), which show opposite trends of AD- and PD-related change to that of the BChE-K variant (rs1803274). However, all of those cohorts were very small, raising the possibility that population differences rather than disease links are involved. Also, BCHE-K’s prevalence showed no correlation with PD, although 10 of the 67 PD patients carried all three BCHE mutations, and in spite of the fact that the BCHE-K variant becomes a significant risk factor for PD in pesticide-exposed individuals (Rösler et al., 2018). Moreover, there was no difference in the co-inherited SNP in the 3′-UTR of the ACHE gene. Inherited SNPs in such non-coding regions may alter the levels of relevant microRNA targets, modify neuronal circuits, and increase disease risk (Hanin et al., 2014). Specifically, a SNP in the ACHE 3′-UTR weakens the interaction with the primate-specific miR-608 (Hanin et al., 2014; Soreq, 2015) and could lead to a cascade of downstream effects that alter important cellular signaling pathways.

Multiple brain regions are involved in the cognitive decline and neuron loss in AD (Perluigi et al., 2016), and PD involves deterioration of both motor and non-motor neurotransmitter systems (Gao and Wu, 2016; Holper et al., 2019). Cholinergic neurons produce the majority of brain AChE (Soreq and Seidman, 2001; Barbash et al., 2017), and disruption of cholinergic transmission has long been associated with AD (Giacobini et al., 2022). Compatible with these statements, we found a large decrease in the most abundant AChE mRNA splice variant (Xia et al., 2022), AChE-S, in the superior temporal gyrus from AD brains and a trend of decrease in the substantia nigra of brains from PD patients compared to controls. Moreover, AChE’s enzyme activity was significantly lower in the superior temporal gyrus of AD patients, and the same tendency was observed in the substantia nigra of PD patients, possibly reflecting loss of neurons in these brain regions.

Previous research has shown that the amyloid-beta (Aβ) peptide and its precursor protein (APP) act as pathogenic triggers in AD, driving the activation of numerous cell signaling pathways such as necrosis, apoptosis, and autophagy, and inevitably leading to neuronal cell death (Leong et al., 2020). The hydrophilic AChE-R variant is amplified during stress-induced apoptosis (Vaknine and Soreq, 2020). Consistent with the stress and anxiety associated with AD and PD progression, AChE-R may initially contribute to limiting the acute, stress-related period of AD and PD (Kaufer et al., 1998). Correspondingly, AChE-R mRNA levels were elevated in the superior temporal gyrus from AD brains and in the stress-associated amygdala of PD brains, whereas the substantia nigra of PD showed no change compared to corresponding controls in spite of the neuronal loss in this region.

The AChE-Next variant harbors an extended N-terminus generated by an alternative upstream promoter that correlates with apoptosis (Toiber et al., 2008). When linked to the AChE-Next domain, a monomeric AChE-R protein could significantly modulate diverse neurological features (Meshorer and Soreq, 2006). A recent study found increases in the AChE-R and AChE-Next variants in the frontal cortex of AD patients compared to controls (Campanari et al., 2016). In comparison, we observed unchanged AChE-Next mRNA levels in the superior temporal gyrus of AD brains and in the substantia nigra of PD brains, but an elevation in the amygdala of PD compared with control brains.

Butyrylcholinesterase plays a physiological role in brain homeostasis (Brimijoin et al., 2016) and may also play a role in the pathogenesis of neurodegenerative diseases (DeBay et al., 2017; Rösler et al., 2018). Correspondingly, BChE levels correlate with the accumulation of neurofibrillary tangles and amyloid plaques (Gómez-Ramos and Moran, 1997), suggesting that it may be an AD modulator (Podoly et al., 2009). Compatible with this prediction, we found elevated BChE mRNA levels in the superior temporal gyrus of AD patients compared to controls. In contrast, PD brains showed no difference compared with control brains.

The cholinergic tone in the brain is affected by the shift between different AChE variants in neurons. Based on the model system of cholinergic differentiation in human-derived neuroblastoma cells, we expected to observe neuronal upregulation of AChE-S and AChE-R but no change in BChE mRNA transcripts. However, oxidative stress conditions led to decreases in all of the AChE and BChE mRNA transcripts, while an antioxidant led to inverse changes in AChE mRNA level, demonstrating that the stress-induced post-transcriptional regulation of cholinesterase transcripts can be rescued and counteracted by neuroprotective therapeutics. Fine-tuning of the ratio between AChE variants may sustain the cholinergic tone and define the balance between neurodamage and neuroprotection in cholinergic neurons.

Our analysis of specific brain regions from AD and PD patients, genotyping datasets, and cell culture experiments all indicate causative disease-specific links with neurodegenerative processes for cholinesterase SNPs and splice variants. Stress stimuli can trigger overexcitation via ACh release, and the feedback reactions can lead to AChE overexpression that may serve to restore normal cholinergic neurotransmission (Sternfeld et al., 1998; Lane et al., 2004). The homeostasis AChE-R/AChE-S ratio may change rapidly in response to environmental factors, supporting the notion that chronic cholinergic imbalances may mediate dysfunction (Lane et al., 2004). RNA-therapeutic modulation of these mRNA variants could involve both brain- and variant-specific approaches. Further research in larger cohorts is needed to validate the role of the cholinergic system in different brain regions, to elucidate the mechanisms underlying AD and PD amelioration, and to develop new therapeutic strategies for AD and PD by regulating cholinergic signaling.

## Data availability statement

The original contributions presented in this study are included in the article/Supplementary material, further inquiries can be directed to the corresponding author.

## Ethics statement

The studies involving human participants were reviewed and approved by the Ethics Committee of The Hebrew University of Jerusalem, Netherlands Brain Bank (NBB) and the Rush University Medical Center Institutional Review Board. The patients/participants provided their written informed consent to participate in this study.

## Author contributions

EB, DG, and HS contributed to the conception and design of the study. MG, TZ, and EB carried out experimental work. NM and MG performed the statistical analysis. MG and HS wrote the first draft of the manuscript. MG, NM, TZ, DG, and HS wrote the sections of the manuscript. All authors contributed to the manuscript revision, read, and approved the submitted version.
