# Supplementary material for: Altered levels of variant cholinesterase transcripts contribute to the imbalanced cholinergic signaling in Alzheimer’s and Parkinson’s disease
Source: Front Mol Neurosci. 2022 Sep 2;15:941467. doi: 10.3389/fnmol.2022.941467 (PMC9479005; doi:10.3389/fnmol.2022.941467)
Supplement: Supplementary file 1 [file Data_Sheet_1.pdf]

Altered levels of variant cholinesterase transcripts contribute to the imbalanced cholinergic signaling in Alzheimer's and Parkinson's Disease

Muslum Gok[1,2], Nimrod Madrer[2,3], Tamara Zorbaz[2,4], Estelle R. Bennett[2,3], David Greenberg[2,3], David A. Bennett[5], Hermona Soreq[2,3*]

[1]Department of Biochemistry, Faculty of Medicine, Mugla Sitki Kocman University, Mugla, Turkey

[2]Edmond and Lily Safra Center for Brain Sciences, The Hebrew University of Jerusalem, Jerusalem, Israel

[3]Department of Biological Chemistry, The Hebrew University of Jerusalem, Jerusalem, Israel

[4]Biochemistry and Organic Analytical Chemistry Unit, The Institute of Medical Research and Occupational Health, Zagreb, Croatia

[5]Department of Neurological Sciences, Rush University Medical Center, Chicago, IL, USA

*** Correspondence:** Hermona Soreq, hermona.soreq@mail.huji.ac.il

Content

Supplementary Table S1. Clinical data and genotyping results for human post-mortem brain tissues provided by the Netherland Brain Bank for Parkinson’s disease with dementia and non-demented control patients.

Supplementary Table S2. Clinical data and genotyping results for human post-mortem brain tissue from the Rush University for Alzheimer's disease and control patients.

Supplementary Table S3. All qPCR primers used in the study.

Supplementary Figure S1. Cholinergic differentiation of human neuroblastoma cell lines induced by 4 days treatment with 100 ng/mL CNTF and 10 μM ATRA.

Supplementary Table S1. Clinical data and genotyping results for human post-mortem brain tissues provided by the Netherland Brain Bank for Parkinson's disease with dementia (PD) and non-demented control (CTRL) patients. Asterisk next to SampleID (*) signifies carriers of all three SNPs of BChE.

Sample ID	Autopsy ID	Region	Sex	Age	ApoE	BCHE-K (rs1803274)	BCHE-Next (rs1126680)	Intron2 (rs55781031)	ACHE-3UTR (rs17228616)	Braak	Diagnosis	Procedures
2	S13/056	Amygdala	m	95	42	N/A	N/A	N/A	N/A	2	CTRL	qPCR
3	S11/090	Amygdala	f	85	NA	N/A	N/A	N/A	N/A	3	CTRL	qPCR
4	S12/042	Amygdala	f	83	44	N/A	N/A	N/A	N/A	2	CTRL	qPCR
5	S12/070	Amygdala	m	79	33	SNP	WT	N/A	WT	2	CTRL	qPCR, SNP Genotyping
6*	S12/104	Amygdala	m	79	32	**SNP**	**SNP**	**SNP**	WT	2	CTRL	qPCR, SNP Genotyping
7	S13/010	Amygdala	f	89	NA	N/A	N/A	N/A	N/A	3	CTRL	qPCR
8	S13/011	Amygdala	f	92	NA	N/A	N/A	N/A	N/A	3	CTRL	qPCR
9	S14/051	Amygdala	m	92	NA	N/A	N/A	N/A	N/A	3	CTRL	qPCR
11	S00/055	Locus Coeruleus	m	82	33	WT	WT	N/A	WT	2	CTRL	SNP Genotyping
14*	*90/098	Locus Coeruleus	m	74	33	**SNP**	**SNP**	**SNP**	WT	0	CTRL	SNP Genotyping
16*	S01/322	Locus Coeruleus	f	73	43	**SNP**	**SNP**	**SNP**	WT	2	CTRL	SNP Genotyping
18	S12/049	Locus Coeruleus	f	70	33	WT	WT	N/A	SNP	2	CTRL	SNP Genotyping
21	S96/251	Locus Coeruleus	m	84	33	WT	WT	N/A	WT	1	CTRL	SNP Genotyping
27	S03/254	Middle temporal gyrus	m	82	43	WT	WT	N/A	WT	4	CTRL	SNP Genotyping
28	S05/269	Middle temporal gyrus	m	87	33	WT	WT	N/A	WT	3	CTRL	SNP Genotyping
29	S10/109	Middle temporal gyrus	f	60	32	WT	WT	N/A	WT	1	CTRL	SNP Genotyping
34	S14/029	Middle temporal gyrus	f	78	NA	WT	WT	N/A	WT	1	CTRL	SNP Genotyping
35	S14/043	Middle temporal gyrus	f	60	NA	WT	WT	N/A	WT	0	CTRL	SNP Genotyping
37	S01/054	Substantia Nigra	m	77	32	WT	WT	N/A	WT	1	CTRL	qPCR, SNP Genotyping
38	S96/206	Substantia Nigra	f	70	33	WT	WT	N/A	WT	1	CTRL	qPCR, SNP Genotyping

39	S98/059	Substantia Nigra	f	69	33	WT	WT	N/A	WT	1	CTRL	qPCR, SNP Genotyping
40	*92/001	Substantia Nigra	m	83	33	WT	WT	N/A	SNP	1	CTRL	qPCR, SNP Genotyping
42	S97/366	Substantia Nigra	f	77	33	WT	WT	N/A	WT	1	CTRL	qPCR, SNP Genotyping
43	S13/056	Substantia Nigra	m	95	42	WT	WT	N/A	WT	2	CTRL	qPCR, SNP Genotyping
44	S09/039	Substantia Nigra	m	78	33	WT	WT	N/A	SNP	1	CTRL	qPCR, SNP Genotyping
45*	S10/035	Substantia Nigra	f	73	44	**SNP**	**SNP**	**SNP**	WT	1	CTRL	qPCR, SNP Genotyping
46*	S11/090	Substantia Nigra	f	85	NA	**SNP**	**SNP**	**SNP**	WT	3	CTRL	qPCR, SNP Genotyping
47	S11/096	Substantia Nigra	f	70	33	WT	WT	N/A	WT	2	CTRL	qPCR, SNP Genotyping
48	S12/042	Substantia Nigra	f	83	44	WT	WT	N/A	WT	2	CTRL	qPCR, SNP Genotyping
49	S13/010	Substantia Nigra	f	89	NA	WT	WT	N/A	WT	3	CTRL	qPCR, SNP Genotyping
50*	S13/011	Substantia Nigra	f	92	NA	**SNP**	**SNP**	**SNP**	WT	3	CTRL	qPCR, SNP Genotyping
51	S13/016	Substantia Nigra	m	83	33	WT	WT	N/A	WT	1	CTRL	qPCR, SNP Genotyping
52	S14/051	Substantia Nigra	m	92	NA	SNP	WT	N/A	WT	3	CTRL	qPCR, SNP Genotyping
54	S09/195	Amygdala	m	69	33	N/A	N/A	N/A	N/A	1	PD	qPCR
55	S09/207	Amygdala	f	67	33	N/A	N/A	N/A	N/A	1	PD	qPCR
56	S09/235	Amygdala	m	70	32	N/A	N/A	N/A	N/A	1	PD	qPCR
58	S10/313	Amygdala	m	61	33	N/A	N/A	N/A	N/A	1	PD	qPCR
59	S11/026	Amygdala	m	77	43	N/A	N/A	N/A	N/A	3	PD	qPCR
60	S11/062	Amygdala	m	65	33	N/A	N/A	N/A	N/A	1	PD	qPCR
61	S12/011	Amygdala	m	73	33	N/A	N/A	N/A	N/A	3	PD	qPCR
62	S12/013	Amygdala	m	76	33	N/A	N/A	N/A	N/A	3	PD	qPCR
63	S12/074	Amygdala	m	87	43	N/A	N/A	N/A	N/A	3	PD	qPCR
64	S12/076	Amygdala	f	81	33	N/A	N/A	N/A	N/A	3	PD	qPCR
65	S12/080	Amygdala	m	86	32	N/A	N/A	N/A	N/A	3	PD	qPCR

67	S13/087	Amygdala	m	80	NA	N/A	N/A	N/A	N/A	1	PD	qPCR
69	S14/009	Amygdala	f	83	NA	N/A	N/A	N/A	N/A	2	PD	qPCR
70	S14/022	Amygdala	m	77	NA	N/A	N/A	N/A	N/A	2	PD	qPCR
71	S13/086	Amygdala	m	81	NA	N/A	N/A	N/A	N/A	2	PD	qPCR
72	S14/032	Amygdala	m	83	NA	N/A	N/A	N/A	N/A	2	PD	qPCR
73	S14/076	Amygdala	m	78	NA	N/A	N/A	N/A	N/A	3	PD	qPCR
74	S13/088	Amygdala	f	71	NA	N/A	N/A	N/A	N/A	2	PD	qPCR
118	S13/027	Substantia Nigra	m	76	NA	WT	WT	WT	WT	2	PD	qPCR, SNP Genotyping
119	S13/041	Substantia Nigra	m	70	NA	WT	WT	WT	WT	1	PD	qPCR, SNP Genotyping
120	S09/195	Substantia Nigra	m	69	33	WT	WT	WT	WT	1	PD	qPCR, SNP Genotyping
121	S09/235	Substantia Nigra	m	70	32	WT	WT	WT	WT	1	PD	qPCR, SNP Genotyping
122	S09/207	Substantia Nigra	f	67	33	SNP	WT	WT	WT	1	PD	qPCR, SNP Genotyping
123	S13/088	Substantia Nigra	f	71	NA	WT	WT	N/A	SNP	2	PD	qPCR, SNP Genotyping
124	S10/313	Substantia Nigra	m	61	33	SNP	WT	WT	SNP	1	PD	qPCR, SNP Genotyping
125*	S11/062	Substantia Nigra	m	65	33	**SNP**	**SNP**	**SNP**	WT	1	PD	qPCR, SNP Genotyping
126	S11/026	Substantia Nigra	m	77	43	WT	WT	WT	WT	3	PD	qPCR, SNP Genotyping
127	S12/011	Substantia Nigra	m	73	33	WT	WT	N/A	WT	3	PD	qPCR, SNP Genotyping
128	S12/013	Substantia Nigra	m	76	33	SNP	SNP	WT	WT	3	PD	qPCR, SNP Genotyping
129	S12/074	Substantia Nigra	m	87	43	SNP	WT	SNP	WT	3	PD	qPCR, SNP Genotyping
130	S12/076	Substantia Nigra	f	81	33	SNP	WT	WT	WT	3	PD	qPCR, SNP Genotyping
131	S12/080	Substantia Nigra	m	86	32	SNP	WT	WT	WT	3	PD	qPCR, SNP Genotyping
133	S13/087	Substantia Nigra	m	80	NA	WT	WT	N/A	WT	1	PD	qPCR, SNP Genotyping
135	S14/009	Substantia Nigra	f	83	NA	SNP	WT	WT	WT	2	PD	qPCR, SNP Genotyping
136	S13/086	Substantia Nigra	m	81	NA	WT	WT	N/A	WT	2	PD	qPCR, SNP Genotyping

137	S14/022	Substantia Nigra	m	77	NA	WT	SNP	WT	WT	2	PD	qPCR, SNP Genotyping
138	S14/032	Substantia Nigra	m	83	NA	SNP	WT	N/A	SNP	2	PD	qPCR, SNP Genotyping
139*	S14/076	Substantia Nigra	m	78	NA	**SNP**	**SNP**	**SNP**	WT	3	PD	qPCR, SNP Genotyping
140	S93/166	Amygdala	m	76	43	WT	WT	WT	WT	2	CTRL	qPCR, SNP Genotyping
141	S93/272	Amygdala	m	80	43	WT	WT	WT	WT	0	CTRL	qPCR, SNP Genotyping
144	S95/110	Amygdala	m	75	33	WT	WT	WT	WT	2	CTRL	qPCR, SNP Genotyping
146	S96/148	Amygdala	f	71	43	SNP	WT	WT	SNP	2	CTRL	qPCR, SNP Genotyping
147	S97/133	Amygdala	m	68	33	WT	WT	N/A	WT	2	CTRL	qPCR, SNP Genotyping
148	S12/052	Amygdala	f	64	32	SNP	WT	WT	WT	0	CTRL	qPCR, SNP Genotyping
149	S97/270	Substantia Nigra	m	80	33	WT	WT	N/A	WT	0	CTRL	qPCR, SNP Genotyping
150	S97/333	Substantia Nigra	m	79	33	SNP	WT	SNP	SNP	1	CTRL	qPCR, SNP Genotyping
152*	S99/188	Substantia Nigra	m	92	33	**SNP**	**SNP**	**SNP**	WT	2	CTRL	qPCR, SNP Genotyping
153	S99/214	Substantia Nigra	m	79	33	WT	WT	WT	WT	1	CTRL	qPCR, SNP Genotyping
154	S99/216	Substantia Nigra	m	69	33	WT	WT	N/A	WT	1	CTRL	qPCR, SNP Genotyping
155	S00/034	Substantia Nigra	m	78	43	WT	WT	N/A	WT	1	CTRL	SNP Genotyping
156	S88/237	Amygdala	f	81	43	SNP	WT	SNP	WT	NA	PD	SNP Genotyping
158	S91/234	Amygdala	f	70	33	WT	WT	N/A	WT	0	PD	SNP Genotyping
159	*93/088	Amygdala	m	81	32	SNP	WT	WT	WT	NA	PD	SNP Genotyping
160	*93/153	Amygdala	m	81	33	WT	WT	N/A	SNP	NA	PD	SNP Genotyping
161	S93/334	Amygdala	m	73	33	SNP	WT	N/A	WT	0	PD	SNP Genotyping
162	S94/062	Amygdala	f	83	33	WT	SNP	N/A	WT	2	PD	SNP Genotyping
163*	S12/012	Amygdala	f	73	43	**SNP**	**SNP**	**SNP**	WT	2	PD	SNP Genotyping
164	S12/026	Amygdala	f	91	32	SNP	WT	WT	WT	1	PD	SNP Genotyping

Supplementary Table S2. Clinical data and genotyping results for human post-mortem brain tissue from the Rush University for Alzheimer's disease and control patients. Groups are divided by cognitive status (no cognitive impairment-NCI, mild cognitive impairment-MCI, dementia-DEM) and by three stages of Braak scores (LOW- Braak 1/2, MEDIUM-Braak 3/4, and HIGH-Braak 5/6). Asterisk next to SampleID (*) signifies carriers of all three SNPs of BChE.

Sample ID	Region	Sex	ApoE	Braak	BCHE-K (rs1803274)	BCHE -Next (rs1126680)	Intron 2 (rs55781031)	ACHE -3UTR (rs17228616)	Group	Procedures
A61	superior temporal gyrus	f	23	1	SNP	WT	SNP	WT	NCI-LOW	qPCR, SNP Genotyping
B48	superior temporal gyrus	f	34	1	WT	SNP	WT	SNP	NCI-LOW	qPCR, SNP Genotyping
B2	superior temporal gyrus	m	34	2	SNP	WT	WT	WT	NCI-LOW	qPCR, SNP Genotyping
A59	superior temporal gyrus	f	33	1	WT	WT	WT	SNP	NCI-LOW	qPCR, SNP Genotyping
A44	superior temporal gyrus	f	33	2	WT	WT	WT	WT	NCI-LOW	qPCR, SNP Genotyping
B55	superior temporal gyrus	f	33	2	WT	WT	WT	WT	NCI-LOW	qPCR, SNP Genotyping
B18	superior temporal gyrus	m	33	1	wt	WT	WT	N/A	NCI-LOW	qPCR, SNP Genotyping
B5	superior temporal gyrus	m	33	1	WT	WT	WT	N/A	NCI-LOW	qPCR, SNP Genotyping
A31	superior temporal gyrus	m	23	1	SNP	WT	WT	WT	NCI-LOW	qPCR, SNP Genotyping
B40	superior temporal gyrus	f	23	1	WT	WT	WT	WT	NCI-LOW	qPCR, SNP Genotyping
A23	superior temporal gyrus	m	33	2	WT	WT	N/A	WT	NCI-LOW	qPCR, SNP Genotyping
A9	superior temporal gyrus	m	33	2	WT	WT	N/A	WT	NCI-LOW	qPCR, SNP Genotyping
B53	superior temporal gyrus	f	33	1	WT	WT	N/A	N/A	NCI-LOW	qPCR, SNP Genotyping
B54	superior temporal gyrus	f	23	2	WT	WT	N/A	SNP	NCI-LOW	qPCR, SNP Genotyping
A64	superior temporal gyrus	f	33	2	N/A	N/A	N/A	N/A	NCI-LOW	qPCR, SNP Genotyping
A2	superior temporal gyrus	m	34	4	SNP	WT	N/A	N/A	NCI-MED	SNP Genotyping
A48	superior temporal gyrus	f	34	3	SNP	WT	N/A	N/A	NCI-MED	SNP Genotyping
A33	superior temporal gyrus	m	34	3	WT	WT	N/A	N/A	NCI-MED	SNP Genotyping
A54	superior temporal gyrus	f	33	3	SNP	WT	N/A	N/A	NCI-MED	SNP Genotyping

A10	superior temporal gyrus	m	33	4	WT	WT	N/A	N/A	NCI-MED	SNP Genotyping
A65	superior temporal gyrus	f	23	3	WT	WT	N/A	N/A	NCI-MED	SNP Genotyping
A34	superior temporal gyrus	m	33	5	SNP	WT	SNP	N/A	NCI-HIGH	SNP Genotyping
A4	superior temporal gyrus	m	33	5	WT	SNP	WT	N/A	NCI-HIGH	SNP Genotyping
A68	superior temporal gyrus	f	34	5	WT	WT	WT	N/A	NCI-HIGH	SNP Genotyping
A36	superior temporal gyrus	f	33	5	SNP	WT	WT	N/A	NCI-HIGH	SNP Genotyping
A39	superior temporal gyrus	f	33	5	WT	WT	WT	N/A	NCI-HIGH	SNP Genotyping
A15	superior temporal gyrus	m	33	5	WT	WT	N/A	N/A	NCI-HIGH	SNP Genotyping
A55*	superior temporal gyrus	f	33	2	**SNP**	**SNP**	**SNP**	N/A	MCI-LOW	SNP Genotyping
A1	superior temporal gyrus	m	33	1	WT	SNP	N/A	N/A	MCI-LOW	SNP Genotyping
A27	superior temporal gyrus	m	33	2	WT	WT	N/A	N/A	MCI-LOW	SNP Genotyping
A56	superior temporal gyrus	f	33	1	WT	WT	N/A	N/A	MCI-LOW	SNP Genotyping
A26	superior temporal gyrus	m	23	2	WT	WT	N/A	N/A	MCI-LOW	SNP Genotyping
A62	superior temporal gyrus	f	23	2	WT	WT	N/A	N/A	MCI-LOW	SNP Genotyping
A21	superior temporal gyrus	m	34	4	WT	WT	N/A	N/A	MCI-MED	SNP Genotyping
A7	superior temporal gyrus	m	34	4	WT	WT	N/A	N/A	MCI-MED	SNP Genotyping
A37	superior temporal gyrus	f	33	4	WT	WT	N/A	N/A	MCI-MED	SNP Genotyping
A35	superior temporal gyrus	m	23	3	WT	WT	N/A	N/A	MCI-MED	SNP Genotyping
A46	superior temporal gyrus	f	34	3	WT	N/A	N/A	N/A	MCI-MED	SNP Genotyping
A51	superior temporal gyrus	f	33	4	WT	N/A	N/A	N/A	MCI-MED	SNP Genotyping
A16*	superior temporal gyrus	m	24	5	**SNP**	**SNP**	**SNP**	SNP	MCI-HIGH	SNP Genotyping
A40	superior temporal gyrus	f	34	5	N/A	WT	N/A	N/A	MCI-HIGH	SNP Genotyping
A45	superior temporal gyrus	f	34	5	N/A	WT	N/A	N/A	MCI-HIGH	SNP Genotyping
A71	superior temporal gyrus	f	33	5	N/A	WT	N/A	N/A	MCI-HIGH	SNP Genotyping

A13	superior temporal gyrus	m	34	5	WT	N/A	N/A	N/A	MCI-HIGH	SNP Genotyping
A30	superior temporal gyrus	m	33	5	WT	N/A	N/A	N/A	MCI-HIGH	SNP Genotyping
B31*	superior temporal gyrus	m	34	1	**SNP**	**SNP**	**SNP**	WT	DEM-LOW	SNP Genotyping
B1*	superior temporal gyrus	m	0	1	**SNP**	**SNP**	**SNP**	WT	DEM-LOW	SNP Genotyping
A25	superior temporal gyrus	m	34	1	SNP	WT	N/A	N/A	DEM-LOW	SNP Genotyping
B70	superior temporal gyrus	f	34	2	WT	WT	N/A	N/A	DEM-LOW	SNP Genotyping
A14	superior temporal gyrus	m	33	2	SNP	WT	N/A	N/A	DEM-LOW	SNP Genotyping
A52	superior temporal gyrus	f	33	1	SNP	WT	N/A	N/A	DEM-LOW	SNP Genotyping
B35	superior temporal gyrus	m	33	1	SNP	WT	N/A	N/A	DEM-LOW	SNP Genotyping
B58	superior temporal gyrus	f	33	2	SNP	WT	N/A	N/A	DEM-LOW	SNP Genotyping
B64	superior temporal gyrus	f	33	1	SNP	WT	N/A	N/A	DEM-LOW	SNP Genotyping
B66	superior temporal gyrus	f	33	2	SNP	WT	N/A	N/A	DEM-LOW	DEM-LOW
A17	superior temporal gyrus	m	33	1	WT	WT	N/A	N/A	DEM-LOW	SNP Genotyping
B15	superior temporal gyrus	m	33	2	WT	WT	N/A	N/A	DEM-LOW	SNP Genotyping
B39	superior temporal gyrus	f	33	2	WT	WT	N/A	N/A	DEM-LOW	SNP Genotyping
B67	superior temporal gyrus	f	33	2	WT	WT	N/A	N/A	DEM-LOW	SNP Genotyping
B32	superior temporal gyrus	m	24	1	WT	WT	N/A	N/A	DEM-LOW	SNP Genotyping
A41	superior temporal gyrus	f	23	2	WT	WT	N/A	N/A	DEM - LOW	SNP Genotyping
A49	superior temporal gyrus	f	23	2	WT	WT	N/A	N/A	DEM - LOW	SNP Genotyping
A28	superior temporal gyrus	m	23	2	WT	N/A	N/A	N/A	DEM - LOW	SNP Genotyping
A38	superior temporal gyrus	f	34	4	WT	WT	N/A	N/A	DEM - MED	SNP Genotyping
A66	superior temporal gyrus	f	34	4	WT	WT	N/A	N/A	DEM - MED	SNP Genotyping
A8	superior temporal gyrus	m	33	3	SNP	WT	N/A	N/A	DEM - MED	SNP Genotyping
A20	superior temporal gyrus	m	24	3	WT	WT	N/A	N/A	DEM - MED	SNP Genotyping

A47	superior temporal gyrus	f	24	4	WT	WT	N/A	N/A	DEM - MED	SNP Genotyping
B50*	superior temporal gyrus	f	33	5	**SNP**	**SNP**	**SNP**	WT	DEM - HIGH	qPCR, SNP Genotyping
B56*	superior temporal gyrus	f	23	5	**SNP**	**SNP**	**SNP**	WT	DEM - HIGH	qPCR, SNP Genotyping
B38	superior temporal gyrus	f	44	5	SNP	WT	WT	WT	DEM - HIGH	qPCR, SNP Genotyping
A58	superior temporal gyrus	f	34	5	SNP	WT	WT	WT	DEM - HIGH	qPCR, SNP Genotyping
B49	superior temporal gyrus	f	34	5	SNP	WT	WT	WT	DEM - HIGH	qPCR, SNP Genotyping
A11	superior temporal gyrus	m	34	5	WT	WT	WT	SNP	DEM - HIGH	qPCR, SNP Genotyping
A50	superior temporal gyrus	f	34	5	WT	WT	WT	WT	DEM - HIGH	qPCR, SNP Genotyping
B42	superior temporal gyrus	f	34	5	WT	WT	WT	WT	DEM - HIGH	qPCR, SNP Genotyping
B19	superior temporal gyrus	m	33	5	SNP	WT	WT	SNP	DEM - HIGH	qPCR, SNP Genotyping
B14	superior temporal gyrus	m	33	5	SNP	WT	WT	WT	DEM - HIGH	qPCR, SNP Genotyping
A3	superior temporal gyrus	m	33	5	WT	WT	WT	WT	DEM - HIGH	qPCR, SNP Genotyping
A12	superior temporal gyrus	m	33	5	WT	WT	N/A	SNP	DEM - HIGH	qPCR, SNP Genotyping
A43	superior temporal gyrus	f	33	5	WT	WT	N/A	SNP	DEM - HIGH	qPCR, SNP Genotyping
B3	superior temporal gyrus	m	24	5	WT	WT	N/A	WT	DEM - HIGH	qPCR, SNP Genotyping
A57	superior temporal gyrus	f	33	5	WT	N/A	N/A	WT	DEM - HIGH	qPCR, SNP Genotyping
A32	superior temporal gyrus	m	33	5	N/A	N/A	N/A	N/A	DEM - HIGH	qPCR, SNP Genotyping

Supplementary Table S3. All qPCR primers used in the study.

Gene	Forward/Reverse	Primer Sequence (5'→3')
Butyrylcholinesterase (BCHE)	BCHE-f	GCTCAACAATGTCGATTCTG
	BCHE-r	ATGTAATTGTTCCAGCGATG
ACHE-"Synaptic" (ACHE-S)	ACHE-S-f	CTTCCTCCCCAAATTGCTC
	ACHE-S-r	TCCTGCTTGCTGTAGTGGTC
ACHE-'Readthrough' (ACHE-R)	ACHE-R-f	CTTCCTCCCCAAATTGCTC
	ACHE-R-r	GAAGAGAGGGGTTACACCTGG
ACHE-"N-terminus extended" (ACHE-Next)	ACHE-Next-f	GAATGCTAGGCCTGGTGAT
	ACHE-Next-r	GCAGTGGAAACTTCTGGAAC
Glyceraldehyde-3-phosphate dehydrogenase (GAPDH)	GAPDH-f	CTCAAGATCATCAGCAATGC
	GAPDH-r	GGTCATGAGTCCTTCCACG
BCHE-Intron2 (rs55781031)	BCHE-Intron2-f	CATAGTGGTTCTCAGTGCTC
	BCHE-Intron2-r	ACTGTTCACAAGAGCTTTAAAG
BCHE-K (rs1803274)	BCHE-K-f	GCTCAACAATGTCGATTCTG
	BCHE-K-r	ATGTAATTGTTCCAGCGATG
BCHE-Next (rs1126680)	BCHE-Next-f	CAATTTACAGGCTGGAGCAG
	BCHE-Next-r	TCTCATCCCACAGAATGAGC
ACHE-3UTR (rs17228616)	ACHE-3UTR-f	ACCACTACAGCAAGCAGGAT
	ACHE-3UTR-r	TGAGACATGCAGAGGACCG

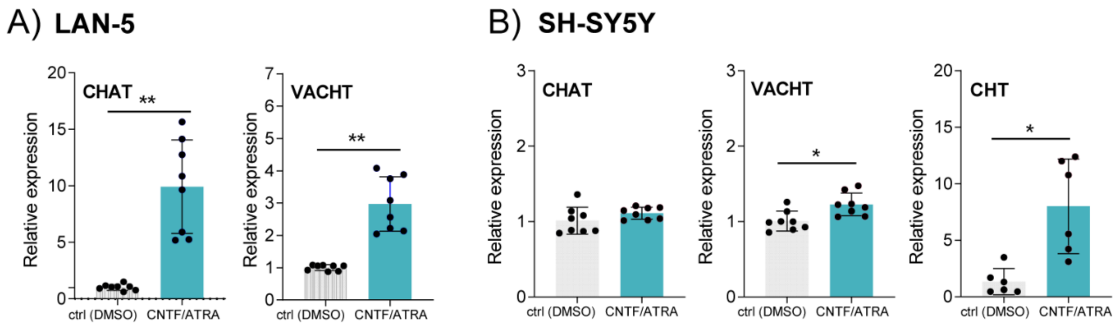

Supplementary figure S1. Cholinergic differentiation of human neuroblastoma cell lines induced by 4 days treatment with 100 ng/mL CNTF and 10 µM ATRA. (A) Upregulation of cholinergic markers CHAT and SLC18A3 (VACHT) in LAN-5 ($p<0.01$) and (B) upregulation of cholinergic markers SLC18A3 (VACHT) and SLC5A7 (CHT) in SH-SY5Y cell line 5 ($p<0.05$).
